# Towards evidence-based palliative care in nursing homes in Sweden: a qualitative study informed by the organizational readiness to change theory

**DOI:** 10.1186/s13012-017-0699-0

**Published:** 2018-01-04

**Authors:** Per Nilsen, Birgitta Wallerstedt, Lina Behm, Gerd Ahlström

**Affiliations:** 1 Department of Medical and Health Sciences, Division of Community Medicine, Linköping University, SE-581 83 Linköping, Sweden; 2 Department of Health and Care Sciences, Linnaeus University, SE-392 81 Kalmar, Sweden; 3 Department of Health Sciences, Faculty of Medicine, Lund University, PO Box 157, SE-221 00 Lund, Sweden

**Keywords:** Organizational readiness to change, Nursing homes, Palliative care, Leadership, Context

## Abstract

**Background:**

Sweden has a policy of supporting older people to live a normal life at home for as long as possible. Therefore, it is often the oldest, most frail people who move into nursing homes. Nursing home staff are expected to meet the existential needs of the residents, yet conversations about death and dying tend to cause emotional strain. This study explores organizational readiness to implement palliative care based on evidence-based guidelines in nursing homes in Sweden. The aim was to identify barriers and facilitators to implementing evidence-based palliative care in nursing homes.

**Methods:**

Interviews were carried out with 20 managers from 20 nursing homes in two municipalities who had participated along with staff members in seminars aimed at conveying knowledge and skills of relevance for providing evidence-based palliative care. Two managers responsible for all elderly care in each municipality were also interviewed. The questions were informed by the theory of Organizational Readiness for Change (ORC). ORC was also used as a framework to analyze the data by means of categorizing barriers and facilitators for implementing evidence-based palliative care.

**Results:**

Analysis of the data yielded ten factors (i.e., sub-categories) acting as facilitators and/or barriers. Four factors constituted barriers: the staff’s beliefs in their capabilities to face dying residents, their attitudes to changes at work as well as the resources and time required. Five factors functioned as either facilitators or barriers because there was considerable variation with regard to the staff’s competence and confidence, motivation, and attitudes to work in general, as well as the managers’ plans and decisional latitude concerning efforts to develop evidence-based palliative care. Leadership was a facilitator to implementing evidence-based palliative care.

**Conclusions:**

There is a limited organizational readiness to develop evidence-based palliative care as a result of variation in the nursing home staff’s change efficacy and change commitment as well as restrictions in many contextual conditions. There are considerable individual- and organizational-level challenges to achieving evidence-based palliative care in this setting. The educational intervention represents one of many steps towards developing a culture conducive to evidence-based nursing home palliative care.

## Background

Nursing homes provide assistance with activities of daily life, such as maintenance of personal hygiene, preparation of special diets, and administration of medication, for persons who are unable to fully perform such activities without help. Multi-morbidity and cognitive and physical impairments are common reasons for nursing home admission when older people cannot be cared for at home [1]. Nursing home care is commonly provided until their death [2]. However, in spite of the high mortality among the elderly persons, nursing homes have not focused on palliative care, i.e., efforts to relieve suffering and preserve the best possible quality of life until death [3]. Palliative care has traditionally been provided in specialist palliative care clinics to hospitalized patients with advanced-stage cancer [4], yet an aging population in many countries means that an increasing number of people would benefit from palliative care in nursing homes. Palliative care has been defined by the World Health Organization (WHO) as an approach that improves the quality of life of patients and their families facing the problems associated with life-threatening illness, through the prevention and relief of suffering by means of early identification and impeccable assessment and treatment of pain and other problems, physical, psychosocial, and spiritual [5]. WHO has called for initiatives to improve palliative care for older people [5].

Sweden has a policy of supporting older people to live a normal life in their own homes for as long as possible [2]. Therefore, it is often the oldest and most frail individuals who move into nursing homes, which means that a high proportion of all deaths among older people in Sweden occur in nursing homes [6]. Staff in nursing homes will increasingly encounter dying residents and be expected to meet their existential needs. However, studies have shown that nursing home staff tend to avoid conversations about death and dying because such issues cause emotional strain among the staff [7, 8]. The lack of quality palliative care leads to increased suffering among the nursing home residents because of the unmet existential concerns [9, 10].

Recognizing the importance of achieving improved palliative care in nursing homes and other settings, the Swedish National Board of Health and Welfare has formulated goals based on the WHO guidelines for palliative care [11]. The Swedish recommendation is that everyone should have access to evidence-based palliative care when the need arises, regardless of diagnosis, form of care, age, or where in the country a person lives [12]. Nursing homes in Sweden do not have a tradition of providing palliative care. This means that the palliative care in this setting tends to be experience-based, typically relying on the presence of more senior staff members who have encountered dying residents. This “knowing-doing” gap concerning evidence-based palliative care in nursing homes was the impetus for the project Implementation of Knowledge-Based Palliative Care in Nursing Homes (Swedish acronym KUPA). The project was developed by researchers at three universities in Sweden and involved managers, staff, older people, and their relatives at 20 nursing homes. An educational intervention was provided to nursing home staff and managers, addressing knowledge and skills of relevance to develop evidence-based palliative care [13]. This study explores the organizational readiness to implement palliative care in nursing homes in Sweden based on the evidence-based guidelines to support staff. The aim was to identify barriers and facilitators to implementing evidence-based palliative care in the nursing homes.

## Methods

### Study setting

The Swedish welfare system is largely funded by taxes and provides equal access for everyone to health care, elderly care, and social services based on each person’s need of support. The system is characterized by shared welfare responsibility between 21 county councils and 290 municipalities. County councils provide primary and secondary health care, whereas municipalities are responsible for social services and providing health care for older people living at home or in nursing homes.

In nursing homes, the residents live in their own apartments with care provided around the clock. The right to an apartment in a nursing home is based on the older person’s need of everyday care assessed by social workers in the municipality. This typically happens when a person is too sick or frail to be able to manage an independent life at home. Staff in nursing homes consists of assistant nurses (the largest group), registered nurses, occupational therapists, and physiotherapists.

### Study participants

The inclusion criterion for this study was managers working in the nursing homes included in the KUPA project. All managers in the 20 nursing homes who took part in the educational intervention to achieve evidence-based palliative care agreed to participate in this study and were interviewed. The responsible managers in all nursing homes received an information letter about the study from the project leader (GA) and were asked to participate. Thereafter, the managers were contacted by e-mail by the interviewer to set up an interview. The 20 nursing homes were located in seven municipalities in the southern part of Sweden. In addition, two higher-level managers who were each responsible for all elderly care in a municipality were interviewed. They received information and were approached in the same way as the nursing home managers. Information on the interviewed managers is provided in Table 1.

**Table 1:** Managers interviewed for the study (*n* = 22)

Nursing home	Educational background	Age (years)	Years as manager	Number of staff in the nursing home
A	Social worker	56	2.5	32
B	Registered nurse	42	5.5	32
C	Registered nurse	60	25	60
D	Registered nurse	65	25	74
E	Registered nurse	62	28	57
F	Social worker	48	6	44
G	Human resources education	67	10	41
H	Social worker	55	2	51
I	Registered nurse	62	35	34
J	Social worker	50	8.5	34
K	Social worker	58	33	45
L	Several educations	37	1	45
M	Social worker	61	40	60
N	Social worker	42	9	48
O	Social worker, nurse practitioner	58	17	43
P	Sociologist	61	13	110
Q	Social worker, nurse practitioner	55	38	80
R	Social worker	52	19	78
S	Social worker	49	28	38
T	Human resources education	45	3	90
U*	Behavioral science education	49	12	Not applicable
V*	Social worker	49	23	Not applicable

*Higher-level managers who were each responsible for all elderly care in a municipality. All managers were female

The managers acted as key informants for their nursing homes. Key informants are persons with relevant knowledge about the issues investigated [14], i.e., efforts to implement evidence-based palliative care in nursing homes.

### The educational intervention

The educational intervention, i.e., the implementation strategy (also referred to as implementation intervention), intended to facilitate the development of an evidence-based palliative care, consisted of five seminars aimed at conveying knowledge and skills of relevance for providing evidence-based palliative care in the nursing homes. Two Swedish documents were used as the basis for the seminars: a National Palliative Care program by the Regional Co-operative Cancer Centers [15] and a national knowledge support document by the National Board of Health and Welfare [12]. Both documents are based on the WHO definition of palliative care [16] and WHO reports [4, 5]. The seminars combined lecture-style presentations and more interactive group discussions. They were provided as an outreach course and took place in the participating nursing homes. This strategy was considered the most appropriate on the basis of the presumed low level of knowledge among the staff concerning evidence-based palliative care principles.

The seminar content was determined after the discussions with staff, informal caregivers, and patients representing both hospital and community care. The research team developed an educational booklet primarily based on the two knowledge documents [12, 15]. The seminars and accompanying educational booklet addressed five themes: the palliative approach and dignified care, next of kin, existence and dying, symptom relief, and collaborative care. The themes were adjusted somewhat based on the expressed needs and interests at each nursing home. The booklet included recommended assignments to do as preparations before each seminar as well as assignments to complete after each seminar. A list of references for further self-studying was also given in the booklet.

The seminar group at each nursing home consisted of 8–10 participants and met approximately once a month over a period of 6 months. The participants belonged to different professions and had different positions in the nursing homes: manager, assistant nurses, registered nurses, occupational therapists, and physiotherapists. The seminars were led by five registered nurses and researchers (including LB and BW) and one registered nurse who worked clinically, all with experience from working as nurses in palliative and geriatric care settings. The participants were selected by the manager of each nursing home, with the aim of including different professions and some staff members who might continue as seminar leaders for further training of the entire staff at each nursing home. Approximately 200 staff members and 20 nursing home managers participated in the course at 20 nursing homes.

### Data collection

A semi-structured interview guide was developed by the research team behind the project. The guide was tested in one interview with the main purpose of determining whether the interview questions were suitable for obtaining rich answers in relation to the study aims. The questions were found to be clear and informative. Consequently, no revision of the interview guide was done, and the test interview was included in the analysis. The interviews were conducted by two of the authors of the study (LB, BW) and took place from September 2015 to December 2016. The nursing home managers were interviewed in their respective nursing home, whereas the municipality managers were interviewed at the two municipalities’ administrative facilities. The interviews were conducted towards the end of the course, between the fourth and fifth seminars. The interviews lasted between 31 and 65 min, with the average duration being 49 min.

The interviews were based on six overarching questions: What does the palliative care initiative mean for the nursing home(s) you are responsible for?; Do you have a plan for how the change effort will be carried out after the seminars are over, and what are the plans from a longer perspective?; How do you view readiness for change among your staff?; How do you view your staff’s motivation and commitment to the change?; What faith do you believe the staff have in their own ability to achieve change?; and What facilitates and what impedes implementation of palliative care in your nursing home, and how important are various facilitators and barriers in your opinion? Probes and questions asking for clarification were added when needed. At the end of each interview, the interviewer asked if there was anything that had not been elucidated. All interviews were recorded and transcribed verbatim.

### Theoretical framework

The interview questions were informed by the theory of Organizational Readiness for Change (ORC) developed by Weiner [17]. ORC was also used as a framework to analyze the data by means of categorizing barriers and facilitators for implementing evidence-based palliative care in terms of the skills and knowledge addressed in the seminars.

ORC is conceptualized as a “shared team property” among organization members, but it can be assessed at both individual and supra-individual levels (e.g., team, department, or organization) [17: 5]. We used ORC to study the supra-individual level since the implementation of more substantial changes in health care usually requires collective and coordinated actions by many organization members [17]. ORC posits that organization members’ change commitment and change efficacy promote organizational readiness to implement change. The term “readiness” connotes a state of being both psychologically and behaviorally prepared to take action (i.e., willing and able). The theory also describes contextual factors as a determinant of organizational readiness to change because such factors can influence change commitment and change efficacy. The three overarching determinants of ORC are [17] as follows:

- Change efficacy: organization members’ beliefs in their capabilities to execute the planned actions involved in the change. Can they implement this change effectively given the situation they currently face?
- Change commitment: organization members’ resolve to pursue the courses of action involved in change implementation. To what extent do organization members value the specific impending change?
- Context: resources, structure, and culture that influence the organization members’ preparedness to implement the change. How does the context affect the organizational members’ willingness or ability to take action?

### Data analysis

The interviews were analyzed using conventional content analysis in accordance with the procedure described by Hsieh and Shannon [18]. The analysis was done by hand (no software was used). As a first step, all authors read and re-read all transcripts to obtain an understanding of the whole. The next step in the process was led by PN, who highlighted words and sentences in the text that captured various key statements in relation to the study aim. During this process, codes that reflected more than one key statement developed; the codes were then aggregated into clusters based on similarity of the content and their relation to each other. After re-examination, the initial clusters were merged into sub-categories, which were given labels that provided an overall description of their content [18]. The sub-categories were cross-examined to ascertain that they were defined in such a way that they were internally as homogeneous as possible and externally as heterogeneous as possible [19].

In the next step, the findings concerning the contents of the sub-categories were compared and contrasted with the three constructs (i.e., categories) of ORC, using a deductive approach. The sub-categories were mapped onto the three constructs by PN. All authors discussed the contents of the categories using triangulating analysis, i.e., the authors independently analyzed the same data and compared their findings. Discussion about this mapping process continued among all authors until no inconsistencies existed, and a shared understanding was reached in order to prevent researcher bias and strengthen the internal validity [20]. Representative quotes were selected by PN in collaboration with the other authors and translated from Swedish to English by PN before being examined by all authors.

## Results

The analysis of the data yielded ten factors (i.e., sub-categories, in bold type in the text) acting as facilitators and/or barriers to developing evidence-based palliative care in the nursing homes, based on the managers’ statements (Table 2). Quotes from 15 managers (numbered 1–15) are used to illustrate the findings.

**Table 2:** Quality content analysis of interviews with managers (*n* = 22)

Category in ORC	Sub-category	Explanation
Change efficacy	Competence and confidence (F/B)	Competence and confidence have to do with the staff’s experience and knowledge concerning palliative care issues and their beliefs that they can learn the principles of palliative care and develop evidence-based palliative care in the nursing home
	Face dying persons (B)	Facing dying persons concerns the staff’s beliefs in their capabilities to address or handle death and dying persons to be able to develop evidence-based palliative care in the nursing home
Change commitment	Motivation (F/B)	Motivation concerns the staff’s willingness and determination to develop evidence-based palliative care in the nursing home
	Attitudes to work in general (F/B)	Attitudes to work are associated with the staff’s interest and engagement in their work in general of potential relevance for developing evidence-based palliative care in the nursing home
	Attitudes to changes at work (B)	Attitudes to changes at work are related to the staff’s resolve to pursue development of evidence-based palliative care in the nursing homes despite experiencing many concurrent changes at work
Context	Resources (B)	Resources refer to the availability of financial and personnel resources of relevance to developing evidence-based palliative care in the nursing home
	Time (B)	Time has to do with the availability of time to allow the staff to learn the principles of palliative care and develop evidence-based palliative care in the nursing home
	Plans (F/B)	Plans relate to developing structures or concrete strategies for continued efforts to develop evidence-based palliative care in the nursing home
	Leadership (F)	Leadership deals with the influence of the nursing home managers and other leaders on the staff in order to develop evidence-based palliative care in the nursing home
	Decisional latitude (F/B)	Decisional latitude refers to the nursing home managers’ autonomy to make work-related decisions conducive to developing evidence-based palliative care in the nursing home

The three categories of the Organizational Readiness to Change (ORC) theory concern change efficacy, change commitment, and context [17]. The sub-categories act as facilitators (F) and/or barriers (B). F are those that are likely to contribute to or enable development of evidence-based palliative care according to the managers’ statements, whereas B are those factors that can be expected to hinder this development on account of the managers’ descriptions. Some factors functioned as both F and B (listed as F/B)

### Change efficacy

The managers noted that some of the staff members have sufficient *competence* and *confidence* and believe that they can learn and provide evidence-based palliative care to the residents. However, the managers also recognized that other members of the staff would likely need a great deal of support because they were unsure of their abilities. In general, the managers believed that staff who had worked longer were more competent and confident. Therefore, based on the managers’ descriptions, current competence and confidence function both as a facilitator and a barrier to developing evidence-based palliative care.

The biggest obstacle is [lack of] knowledge. (1)

Yes, the [ability] varies, I believe. I think some feel very… that I can do this gallantly, but there are also those who think, ‘Can I do this?’ (2)

I think it [trust in one’s own ability] is mixed. But I think it’s very dependent on how long you’ve worked. (3)

According to the managers, many nursing home staff members express reluctance to *face dying persons*. They do not appreciate talking about death or being too closely involved when older people are dying in the nursing home. Managers noted that such situations tend to be perceived as uncomfortable for some staff who may struggle with what to say or how to act. The managers’ descriptions thus mean that hesitation in addressing or facing death or dying persons constitutes a barrier to developing evidence-based palliative care in the nursing homes.

Some have fear, because it exists. (3)

Especially those [older people] who may have lived here for a while, you have created a relationship with them. Then some may think that it is very difficult when the person becomes palliative and will die. Some think that it becomes too personal. (4)

### Change commitment

The managers believed that the nursing home staff are somewhat mixed with regard to their *motivation* to learn and practice the principles of palliative care. Many staff members appeared to be genuinely eager to develop their knowledge and skills in palliative care, but interest seemed to differ considerably among the staff, according to the managers. Hence, motivation functions as both a facilitator and barrier to developing evidence-based practice in view of the managers’ descriptions.

After all, it’s very individual among the staff, how they commit to this and how they handle it. ... And I think it’s important to try to highlight the staff who have the dedication and knowledge, and the desire to work with this. (5)

I saw when I was out to gauge interest in the education, that it was considerable, so we had to make it a lottery. So there is an interest. ... They want knowledge about it. You just want to be safe and secure [in what you do]. (6)

The managers acknowledged that most of the nursing home staff members are engaged in their work and assume a shared responsibility for providing quality elderly care. The managers considered positive *attitudes to work in general* as an important asset for developing evidence-based palliative care. However, they also noted that there were staff members who were less passionate about their work and would therefore be less inclined to learn or practice the principles of palliative care. Based on the managers’ statements, attitude to work is therefore both a facilitator and barrier to developing evidence-based palliative care.

I’m mighty proud of their commitment. In connection with palliative care, there is often an enormous commitment to the people it concerns. They take great responsibility. (7)

Then there are always exceptions and that’s the way it always is, with everything. There are those who sigh and think that this is futile. (8)

The managers believed that many staff members hold overall negative *attitudes to changes at work*, decreasing their commitment to learning the principles of palliative care. The nursing home managers and staff had experienced many changes over the years. The managers believed there was a risk that the aim to introduce principles of palliative care would meet with limited enthusiasm as a result of a certain “change fatigue” among many staff members, thus functioning as a barrier to the development of evidence-based palliative care.

We have had a lot of changes here. I think they are quite fed up. ... What makes it difficult is all the changes we have made so far and they might be tired of changes or improvement work. (6)

It may happen that you get saturated. And it’s always about engaging and retaining this motivation among the staff. If it becomes too much I think you grow tired. (9)

We can never focus only on one thing because there is so much happening in the rest of the world, which affects us, decisions and changes of laws and everything. That [the changes] may be an obstacle. (10)

### Context

Many managers talked about restricted financial *resources*, which often made it difficult to carry out continuous professional education to achieve quality care for the older people and provide support to the staff in learning and developing new competencies, including palliative care principles. According to the managers’ accounts, limited resources, therefore, are a barrier to developing evidence-based palliative care.

Resources are needed if we are to educate ourselves, if we are to improve ourselves. ... We have a negative [economic] balance and we cannot sit here and have visions about doing many different things. (7)

I don’t want to initiate too much [activities], because I feel that there is so much right now that we have to finish first. I shouldn’t mention the economy, but I have to lie low from time to time. You cannot start a lot of stuff which you could before when we had better economy. (10)

The managers expressed that development of staff competence could not always be prioritized with regard to *time* to the extent they wanted. They recognized that acquiring and practicing knowledge and skills in palliative care will require time. According to the managers, time limitations restricted the staff’s ability to learn the principles of palliative care, thus functioning as a barrier to developing evidence-based palliative care.

I think today everything goes so fast, so it’s never anchored. … And that’s why it isn’t so good either. (11)

In order to make a change, it must take time and how do we find time? Do we have the time? And when do we take it? (12)

Everything has to be so fast and time is an opponent. ... We’re living in the future, we are not here in the present. (13)

The managers described various ambitions regarding *plans* to continue working towards evidence-based palliative care in the form of structures or strategies for the future, including having individuals as change leaders or quality improvement developers. However, there were also managers who admitted that they had not considered how the knowledge and skills acquired by staff members in the seminars should be transferred to colleagues or be maintained over time. Based on the managers’ statements, plans for developing evidence-based palliative care practice, therefore, function both as a facilitator and barrier for developing evidence-based palliative care.

We have something called change leaders who are chosen somehow ... We can use them as sounding boards and I can discuss all kinds of changes with them. (7)

No, there is no plan at all. (14)

*Leadership* was not only associated with the influence of the nursing home managers, but managers also viewed registered nurses as potential role models from which nurse assistants could learn how to talk about existential issues and obtain knowledge about other principles of palliative care. Competent leadership is therefore a facilitator to developing evidence-based palliative care on the basis of the managers’ descriptions.

I think [registered] nurses have a key role in this. ... For the nurse assistants usually do as the nurses do. ... I have a really good nurse. She has competence and she is calm and secure, which is evident to the staff and [the residents’] relatives. (4)

It’s a lot about management. It’s a lot about co-operation between manager and nurse, and how you work with the staff. (5)

The managers opinioned that their *decisional latitude* to pursue work-related initiatives of potential relevance for developing evidence-based palliative care was somewhat restrained because of the need to carry out many top-down actions initiated at higher political and management levels. Still, they believed that they had considerable autonomy to make decisions concerning nursing home activities and goals. Thus, according to the managers’ statements, room for decisions functions as a facilitator or barrier, depending on the mandate to make decisions to support the development of evidence-based palliative care practice.

I have a mandate for our activities here, but I get the decisions from above. … I must implement what I’m told from above, even if I do not like it. (3)

We are a politically controlled organization, so it [a directive] may come from politics, but there are things we can influence at our unit. If you have some ideas that are in line with what we are going to work for, then you can try it. (15)

## Discussion

This study sought to explore readiness to implement palliative care in nursing homes based on the evidence-based guidelines from the perspective of managers. The three overarching categories of ORC provided a framework for the analysis of the interviews [17]. We found the structure and contents of ORC to be relevant for the study, offering a plausible theoretical explanation of the determinants for developing evidence-based palliative care and providing a useful tool to categorize facilitators and barriers to achieving this care. We did not identify any barriers or facilitators that did not fit into the framework.

We found that four of the ten factors identified (i.e., sub-categories) acted primarily as barriers to evidence-based palliative care in the nursing homes. Fear or hesitancy among the staff to face dying persons and talking with them about existential concerns were mentioned by many of the managers interviewed for the study. This finding is consistent with previous research, which has shown that nurses and other health care practitioners often do not know what to say or do when patients or residents experience existential distress, and many feel insufficiently prepared to provide existential care for the dying [7, 21–25]. The need for training in all aspects of spiritual and existential end-of-life care has been emphasized [10, 26].

The fear of facing the reality of death among nursing home residents may be partially attributed to the prevailing culture in Sweden and many western societies, whereby death is a sensitive or even taboo topic [27]. Increased medication and institutionalization of the dying in contemporary society has led to fewer people having experience of dying persons [28, 29]. In consequence, many people today are unprepared for the existential uncertainty associated with dying [30]. The educational intervention, i.e., the seminars, in which the managers that we interviewed participated was intended to convey knowledge and skills to enhance staff confidence and competence to provide palliative care. The seminars allowed for discussion and sharing of experiences concerning dying persons, which appeared to be valued by the participants. Reflection is typically described as a mechanism to translate experience into learning, by examining one’s attitudes, beliefs, and actions, to draw conclusions to enable better responses in the future [31]. However, Penrod [32] argues that feelings of existential uncertainty can never be fully resolved, implying that health care practitioners in palliative care must learn to live with this uncertainty.

Attitudes to changes at work emerged as another barrier to evidence-based palliative care. The managers found that many staff members were tired of changes, which made them less interested in acquiring the new knowledge and skills required to develop evidence-based palliative care. Managers mentioned previous changes that had lacked focus and were unsuccessful, which had created a sense of change fatigue among the staff. The concept of organization change fatigue has been defined as a general sense of apathy or passive resignation by individuals or teams towards organizational changes. It is associated with increased stress and decreased organizational commitment [33, 34]. Our findings suggest that the concept of change fatigue has considerable relevance with regard to implementing the changes required to develop evidence-based palliative care in nursing homes. Change fatigue indicates the importance of forward-thinking coordination of changes to achieve successful implementation of new practices. Somewhat remarkably, timing or coordination of changes is not addressed in many of the determinant frameworks that are widely used in implementation science to identify and categorize factors that have an impact on implementation success [35].

As expected, resources and time both constituted barriers. This is consistent with other studies on efforts to improve palliative care for older people in nursing homes [36–39]. Resources and time are closely linked; limited resources restrict the number of staff per resident, which has an impact on the time available for staff to communicate with the residents, their families, and colleagues, as well as the time that can be devoted to continuous professional education or improvement efforts. The resource and time constraints also apply to health care in general; these two factors have overwhelmingly been identified as barriers to implementing evidence-based practices in various health care settings [40–43].

Leadership stood out as a potentially important facilitator. The importance of leadership for improving palliative care in nursing homes has also been recognized in other studies (e.g., [39, 44]. The managers in our study believed they could provide crucial support for the staff to develop evidence-based palliative care. They also pointed to registered nurses as having an important role as informal leaders for the many assistant nurses employed in nursing homes. A nursing home study in Norway by Sommerbakk et al. [39] suggested that nurses could act as local champions who would tutor colleagues and give the staff ownership of the improvement project. The importance of role models can be explained with reference to social-cognitive theory [45], which posits that self-efficacy, i.e., the belief in one’s capacity to carry out a specific behavior, can be enhanced by means of vicarious experience such as observing someone else performing a task or handling a situation, e.g., addressing existential concerns with an older person.

Leadership is typically conceptualized as involving a process of one person exerting intentional influence over another person or group in order to achieve a certain outcome in a group or organization [46]. It is important, however, to distinguish between leadership and management. The leader sets goals and seeks new ways to work towards the goals, whereas the manager maintains the status quo and organizes, directs, and controls to achieve goals [47]. Not all managers take on the role as leaders, and not all leaders are managers because there can also be informal leaders, i.e., in line with the role attributed to some registered nurses, according to the managers. The relevance of leadership in implementation science has been acknowledged, yet there has been relatively little empirical research on this concept in this field [48].

Leaders have an important role in shaping the culture of a group, unit, profession, organization, or other social group or unit [49]. Culture has been defined as the shared values (important and lasting ideals and preferences for certain behaviors) and norms (beliefs about acceptable behaviors) and assumptions (unspoken beliefs and expectations) among members of a collective [50]. Other studies concerning palliative care in nursing homes have identified the importance of the culture (e.g., [7, 51]). Changing the nursing home culture so that the values, norms, and assumptions support evidence-based palliative care represents a profound challenge as the palliative care principles are transferred from cancer care to more general care settings such as nursing homes. Froggatt [52] has emphasized that a cultural change is needed as education alone is not sufficient to bring about changes in practice to achieve evidence-based palliative care in nursing homes.

Five of the ten factors acted as both facilitators and barriers for evidence-based palliative care based on the managers’ statements. The managers described their staff as being mixed with regard to competence and confidence in their ability and motivation to learn and practice the principles of evidence-based palliative care. This finding is consistent with much implementation research, which points to motivation, competence, and confidence as important determinants of implementation success [53–55].

Attitudes to work in general also emerged as a “mixed” facilitator and barrier. Although the managers emphasized that most of their staff expressed positive work attitudes, they also recognized that there were those who were less enthusiastic about their work and might be less motivated to learn or practice the principles of palliative care. Attitudes to work can be understood in terms of work climate, i.e., patterns of behavior, attitudes, and feelings, that characterize life in an organization or other unit. Whereas, the culture tends to be deep and stable; the climate is more amenable to change [56]. Sommerbakk et al. [39] identified work climate problems as an important barrier to change. They noted that there was a lack of support from colleagues when some of the staff members were given training and responsibilities that took them away from their daily clinical work.

Finally, plans with regard to structures or strategies to continue efforts to develop evidence-based palliative care and the ability to make decisions concerning such efforts also functioned as both a facilitator and barrier based on the managers’ descriptions. The managers were asked to choose participants for the seminars who could continue as seminar leaders for further education of the staff. Such train-the-trainer schemes have shown promise for disseminating various evidence-based principles and knowledge in different settings (e.g., [57–59]). However, not all managers had considered this option and had not identified suitable individuals who might be willing and able to carry on teaching their colleagues.

Some methodological issues must be considered when interpreting the findings. A qualitative approach was chosen because little is known about facilitators and barriers to developing evidence-based palliative care in nursing homes in Sweden. Interviews with managers were considered the most relevant method for collecting information and gaining a deeper understanding of the nursing homes’ organizational readiness to develop an evidence-based palliative care. The barriers and facilitators identified in this study are not intended as an exhaustive list of all possible determinants; other studies may yield different barriers and facilitators or give different priorities to other factors. The results cannot be directly transferred to other nursing homes in Sweden or internationally. Instead of statistical generalization, we sought analytical (also referred to as theoretical) generalization by comparing our findings with other studies on palliative care in nursing home settings and research on implementation of evidence-based practices in general. It was deemed more appropriate to interview all managers instead of sampling some of them. The data analysis confirmed that data saturation was reached before cessation of the interviews. Transcripts were not returned to the participants for comments, and they could not provide feedback on the findings. An important reason for this is that the same participants will be interviewed for a follow-up study 9 months later, and we did not want them to be influenced by the interpretation of this study.

We used the ORC theory as a framework for the study. According to De Dierckx et al. [60], using a preconceived framework runs the risk of prematurely excluding alternative ways of organizing the data that may be more illuminating. However, we used ORC to broadly inform the questions presented to the managers, and it was not applied until the second phase of the data analysis, after the data had first been analyzed inductively to arrive at facilitators and/or barriers (i.e., sub-categories).

The two authors (LB, BW) who conducted the interviews were also part of the team of six persons who led the seminars. This double role could potentially induce bias, but the managers were in an independent position to them. Furthermore, the interviews did not address the educational interventions, focusing instead on if the managers had plans for how the change effort would be carried out, their views concerning the staff’s readiness, commitment and ability to achieve the change, and what might facilitate or impede the change. The interviewers have considerable interview experience and conveyed to the managers that they should respond as truthfully as possible and should not provide answers to please the interviewer.

The managers acted as key informants for their nursing homes. We sought key informants in accordance with the prerequisites for using this approach. Thus, the managers we interviewed had roles that exposed them to the information we sought, had access to the desired information, and had absorbed the information meaningfully, and were willing and able to communicate their knowledge to the interviewer in an intelligible manner [14]. The managers participated in the seminars and were interviewed after the fourth of five seminars in the course, which means that they could be expected to have a good understanding of the meaning and requirements of evidence-based palliative care and the responses of colleagues participating in the course. However, this does not necessarily mean that the managers were fully aware of the attitudes, beliefs, and motivation among all staff members. Leadership emerged as the only factor that did not constitute a barrier to developing evidence-based palliative care. This finding should be interpreted with caution, because the managers effectively spoke on behalf of themselves as leaders. We did not interview staff who participated in the seminars since we focused on the supra-individual level, which is relevant when applying ORC to explore the implementation of more substantial changes that require collective and coordinated actions by many organization members [17]. ORC is conceptualized in terms of being “a psychological state that organization members hold in common” [17: 5]. Accordingly, the aim was to obtain an understanding of the *organizational* readiness for implementing evidence-based palliative care. Interviews with individual staff members could have provided insights into individuals’ beliefs, attitudes, motivation, and other individual-level cognitions, but such an approach would probably have yielded insufficient information about the readiness of the nursing homes as a whole.

The multidisciplinary research team was a strength of the study, because it permitted different perspectives on the issue of palliative care in nursing homes. The team consisted of three (female) nurses with experience in clinical patient work as well as work on miscellaneous issues concerning research on older people (GA, LB, BW) and an experienced implementation researcher (male), who is an economist and organizational analyst (PN). Another strength was the relatively high number of interviews. This allowed us to use quotations from many different managers, which added transparency and trustworthiness to the findings.

The study contributes important knowledge with regard to implementation science. While the concept of ORC has been widely applied to understand changes in various organizations [61], few empirical implementation science studies in health care settings have used Weiner’s theory-based ORC framework [17]. It was found to provide an excellent framework for informing the data collection and analyzing the data. Choosing one theory, model, or framework often means placing weight on some aspects at the expense of others, thus offering only partial understanding. However, ORC was found to be sufficiently broad to allow for a fairly inductive approach. The study findings point to difficulties in changing a practice when it essentially requires a cultural change. Some of the determinants described in the study are well-known across implementation studies in different settings, but the study also identified a unique barrier in the form of fear or hesitancy among the staff to face dying persons. It suggests the considerable challenge of implementing an evidence-based practice when the desired behavior is emotionally charged and may present difficulties even after participation in educational interventions to acquire necessary skills and knowledge. Rafferty et al. [62] suggest that affective aspects of change readiness have been overlooked, arguing that both theoretical and empirical studies support the relevance of the affective element of attitudes since it may negatively influence change efficacy and change commitment. The study also highlighted the importance of the timing of implementing new practices, which could possibly be seen as an underlying explanation for some of the barriers which are often described in implementation studies, e.g., insufficient time and lack of motivation among staff. The timing of implementation is important to address in implementation studies, which tend to investigate one change initiative at a time rather than viewing them in a broader perspective of the many concurrent change initiatives that practitioners typically face.

In terms of implications, the study suggests the relevance of several initiatives that would build on the identified facilitators to increase the organizational readiness for an evidence-based palliative care. Certain staff members could act as local champions for palliative care and provide support for further education and training in palliative care principles. Rogers [63] and many implementation researchers (e.g., Nilsen [35], Greenhalgh et al. [40], and Damschroder et al. [43]) have emphasized the importance of champions in order to support new practices and overcome indifference or resistance concerning a new way of working. Registered nurses may be best suited to take on such a role. Involving them in train-the-trainer schemes would enable a continuation of the educational intervention after the research project has ended [52, 57–59]. They could tutor colleagues in their own nursing homes and possibly visit other nursing homes. Another option is to designate work time for discussion groups where nursing home staff meet to reflect more informally on palliative care issues. Research suggests the importance of scheduling forums that provide legitimacy for reflection [64, 65]. Both train-the-trainer schemes and discussion forums depend on the managers’ leadership and legitimization to give the activities priority, which is required to develop a culture where facing dying residents’ concerns becomes normal and expected among the staff.

## Conclusions

There is a limited organizational readiness to develop evidence-based palliative care because of considerable variation in the nursing home staff’s change efficacy and change commitment as well as restrictions in many contextual conditions. The findings show that there are considerable individual- and organizational-level challenges to achieving evidence-based palliative care in this setting. The educational intervention undertaken by managers and staff to acquire knowledge and skills in palliative care should be considered one of many steps towards developing a culture conducive to evidence-based nursing home palliative care.

## References

[1] Gustavsson M, Liedberg GM, Larsson Ranada Å (2015). Everyday doings in a nursing home—described by residents and staff. Scand J Occup Ther.

[2] Young Y, Kalamaras J, Kelly L, Hornick D, Yucel R (2015). Is aging in place delaying nursing home admission?. J Am Med Dir Assoc.

[3] Beck I, Jakobsson U, Edberg A-K (2014). Applying a palliative care approach in residential care: effects on nurse assistants’ experiences of care provision and caring climate. Scand J Caring Sci.

[4] Davies E, Higginson IJ (2004). Better palliative care for older people.

[5] Hall S, Petkova H, Tsouros A, Constantini M, Higginson I (2011). Palliative care for older people: better practices.

[6] Broad JB, Gott M, Kim H, Boyd M, Chen H, Connolly MJ (2013). Where do people die? An international comparison of the percentage of deaths occurring in hospital and residential aged care settings in 45 populations, using published and available statistics. Int J Public Health.

[7] Tornøe K, Danbolt LJ, Kvigne K, Sörlie V (2015). A mobile hospice nurse teaching team’s experience training care workers in spiritual and existential care for the dying—a qualitative study. BMC Palliat Care.

[8] Åsberg E, Carlsson M (2013). Practical care work and existential issues in palliative care: experiences of nursing assistants. Int J Older People Nursing.

[9] Ersek M, Carpenter JG (2013). Geriatric palliative care in long-term care settings with a focus on nursing homes. J Palliat Med.

[10] Balboni MJ, Sullivan A, Amobi A, Phelps AC, Gorman DP, Zollfrank A (2013). Why is spiritual care infrequent at the end of life? Spiritual care perceptions among patients, nurses and physicians and the role of training. J Clin Oncol.

[11] WPCA (2014). & WHO. Global atlas of palliative care at the end of life.

[12] National Board of Health and Welfare. Nationellt kunskapsstöd för god palliativ vård i livets slutskede – Vägledning, rekommendationer och indikatorer – Stöd för styrning och ledning [The national knowledge support document for good palliative care at the end of life]. Stockholm: National Board of Health and Welfare; 2013 [in Swedish].

[13] Behm L, Wallerstedt B, Persson M, Ahlström G (2017). Tematräffar om palliativ vård - ett sätt att öka kunskapen inom särskilda boenden.

[14] Marshall MN (1996). The key informant technique. Fam Pract.

[15] Regional Co-operative Cancer Centers (2012). Nationellt vårdprogram för palliativ vård 2012–2014.

[16] WHO. WHO definition of palliative care. 2014. http://www.who.int/cancer/palliative/definition/en/. Accessed 26 May 2017.

[17] Weiner BJ (2009). A theory of organizational readiness for change. Implement Sci.

[18] Hsieh HF, Shannon SE (2005). Three approaches to qualitative content analysis. Qual Health Res.

[19] Krippendorf K (2004). Content analysis. An introduction to its methodology.

[20] Patton MQ (2002). Qualitative research and evaluation methods.

[21] Christensen KH (2008). Spiritual care perspectives of Danish registered nurses. J Holist Nurs.

[22] Pesut B, Fowler M, Taylor EJ, Reimer-Kirkham S, Sawatzky R (2008). Conceptualising spirituality and religion for healthcare. J Clin Nurs.

[23] Noble A, Jones C (2010). Getting it right: oncology nurses’ understanding of spirituality. Int J Palliat Nurs.

[24] Beck I, Törnquist A, Broström L, Edberg A-K (2012). Having to focus on doing rather than being—nurse assistants’ experience of palliative care in municipal residential care settings. Int J Nurs Stud.

[25] Udo C (2014). The concept and relevance of existential issues in nursing. Eur J Oncol Nurs.

[26] Holloway M, Adamson S, McSherry W, Swinton J (2011). Spiritual care at the end of life: a systematic review of the literature.

[27] Leclerc BS, Lessard S, Bechennec C, Le Gal E, Benoit S, Bellerose L (2014). Attitudes toward death, dying, end-of-life palliative care, and interdisciplinary practice in long term care workers. J Am Med Dir Assoc.

[28] Kovach CR (2007). Dying in nursing homes. J Gerontol Nurs.

[29] Blondeau D (2009). La différence: condition of exclusion or of reconnaissance?. Nurs Philos.

[30] Rushton C, Halifax J, Dossey B (2007). Being with dying, contemplative practices for compassionate end-of-life care. Am Nurs Today.

[31] Schön DA (1983). The reflective practitioner: how professionals think in action.

[32] Penrod J (2001). Refinement of the concept of uncertainty. J Adv Nurs.

[33] Bernerth JB, Walker JH, Harris SG (2011). Change fatigue. Work Stress.

[34] Turner D-M (2015). Launch lead live: the executive’s guide to preventing resistance and succeeding with organizational change.

[35] Nilsen P (2015). Making sense of implementation theories, models and frameworks. Implement Sci.

[36] Brueckner T, Schumacher M, Schneider N (2009). Palliative care for older people—exploring the views of doctors and nurses from different fields in Germany. BMC Palliative Care.

[37] Lynch T, Clark D, Centeno C, Rocafort J, de Lima L, Filbet M (2010). Barriers to the development of palliative care in Western Europe. Palliat Med.

[38] Davies N, Maio L, van Riet Paap J, Mariani E, Jaspers B, Sommerbakk R (2014). Quality palliative care for cancer and dementia in five European countries: some common challenges. Aging Ment Health.

[39] Sommerbakk R, Haugen DF, Tjora A, Kaasa S, Hjermstad MJ (2016). Barriers to and facilitators for implementing quality improvements in palliative care—results from a qualitative interview study in Norway. BMC Palliat Care.

[40] Greenhalgh T, Robert G, Macfarlane F, Bate P, Kyriakidou O (2004). Diffusion of innovations in service organizations: systematic review and recommendations. Milbank Q.

[41] Cochrane LJ, Olson CA, Murray S, Dupuis M, Tooman T, Hayes S (2007). Gaps between knowing and doing: understanding and assessing the barriers to optimal health care. J Contin Educ Heal Prof.

[42] Nutley SM, Walter I, Davies HT (2007). Using evidence: how research can inform public services.

[43] Damschroder LJ, Aron DC, Keith RE, Kirsh SR, Alexander JA, Lowery JC (2009). Fostering implementation of health services research findings into practice: a consolidated framework for advancing implementation science. Implement Sci.

[44] Håkanson C, Cronfalk BS, Henriksen E, Norberg A, Ternestedt BM, Sandberg J (2015). First-line nursing home managers in Sweden and their views on leadership and palliative care. Open Nurs J.

[45] Bandura A (1986). Social foundations of thought and action a social cognitive theory.

[46] Gill R (2011). Theory and practice of leadership.

[47] Yukl G (2006). Leadership in organizations.

[48] Reichenpfader U, Carlfjord S, Nilsen P (2015). Leadership in evidence-based practice: a systematic review. Leadersh Health Serv.

[49] Schein EH (2010). Organizational culture and leadership.

[50] Bang H (2009). Organisationskultur.

[51] Casey D, Murphy K, Ni Leime A, Larkin P, Payne S, Froggatt KA (2011). Dying well: factors that influence the provision of good end-of-life care for older people in acute and long-stay settings in Ireland. J Clin Nurs.

[52] Froggatt KA (2001). Palliative care and nursing homes: where next?. Palliat Med.

[53] Eccles MP, Hrisos S, Francis J, Kaner EF, Dickinson HO, Beyer F (2006). Do self-reported intentions predict clinicians’ behaviour: a systematic review. Implement Sci.

[54] Godin G, Bélanger-Gravel A, Eccles MP, Grimshaw JM (2008). Healthcare professionals’ intentions and behaviours: a systematic review of studies based on social cognitive theories. Implement Sci.

[55] Nilsen P, Roback K, Broström A, Ellström PE (2012). Creatures of habit: accounting for the role of habit in implementation research on clinical behaviour change. Implement Sci.

[56] Denison DR (1996). What is the difference between organizational culture and organizational climate? A native’s point of view of a decade of paradigm wars. Acad Manag Rev.

[57] Rajapakse BN, Neeman T, Dawson AH (2013). The effectiveness of a ‘train the trainer’ model of resuscitation education for rural peripheral hospital doctors in Sri Lanka. PLoS One.

[58] Yarber L, Brownson CA, Jacob RR, Baker EA, Jones E, Baumann C (2015). Evaluating a train-the-trainer approach for improving capacity for evidence-based decision making in public health. BMC Health Serv Res.

[59] Madah-Amiri D, Clausen T, Lobmaier P (2016). Utilizing a train-the-trainer model for multi-site naloxone distribution programs. Drug Alcohol Depend.

[60] De Dierckx CB, Gastmans C, Bryon E, Denier Y (2012). QUAGOL: a guide for qualitative data analysis. Int J Nurs Stud.

[61] Weiner BJ, Amick H, Lee SY (2008). Conceptualization and measurement of organizational readiness for change: a review of the literature in health services research and other fields. Med Care Res Rev.

[62] Rafferty AE, Jimmieson NL, Armenakis AA (2013). Change readiness: a multilevel review. J Manag.

[63] Rogers E (2003). Diffusion of innovations.

[64] Docherty P, Boud D, Cressey P, Boud D, Cressey P, Docherty P (2006). Lessons and issues for practice and development. Productive reflection at work. Abingdon: Routledge.

[65] Nilsen P, Nordstrom G, Ellstrom PE (2012). Integrating research-based and practice-based knowledge through workplace reflection. J Work Learn.

